# DNA barcoding and hypopygium shape support delimitation of sympatric *Dissomphalus* species (Hymenoptera, Bethylidae) from the Atlantic rainforest

**DOI:** 10.3897/zookeys.959.53737

**Published:** 2020-08-14

**Authors:** Marina Monjardim, Celso O. Azevedo, Valéria Fagundes

**Affiliations:** 1 Departamento de Ciências Biológicas, Centro de Ciências Humanas e Naturais, Universidade Federal do Espírito Santo, 29.075-910, Vitória, Espírito Santo, Brazil

**Keywords:** COI, genetic diversity, molecular systematics, new species, wasps

## Abstract

*Dissomphalus* is a cosmopolitan genus of Bethylidae and has 269 Neotropical species divided into 32 species-groups, mostly defined by the genital and the tergal process structures. *Dissomphalus rectilineus* and *D. concavatus* are sympatric species in the *ulceratus* species-group. Members of the species-group share many similarities in the morphology of the head, hypopygium, tergal process and genitalia, but may be distinguished by the structure of the hypopygium. Previous studies have found intermediate structures of the hypopygium in the sympatric areas and raised questions about the distinctiveness of these two species. We sequenced 340 bp of the mitochondrial gene cytochrome oxidase I of 29 specimens from Brazil and Paraguay, calculated the genetic divergence among specimens, and recovered the phylogenetic relationships between taxa. In addition, we compared the morphology of the hypopygium to evaluate its use as a species-specific diagnostic character using the genetic divergence values. We recovered three well-supported monophyletic groups (intraclade divergence from 1.3 to 13.4%) and three hypopygium morphologies associated with each clade, two of them associated with *D. rectilineus* and *D. concavatus* (as described in the literature); the third one is new, not associated with any known species. The divergence between the *D. rectilineus* and *D. concavatus* clades was 19%, while the third clade is divergent from each species by 19–20%. If fully described, the hypopygium shape associated with the COI sequence will represent an extremely promising approach to the diagnosis of *Dissomphalus* species.

## Introduction

*Dissomphalus* Ashmead, 1893 (Pristocerinae) is the most species-rich genus of Bethylidae currently comprising 424 species worldwide (Azevedo et al. 2018). In the Neotropical region, almost 270 species of *Dissomphalus* were organized into 32 species -groups defined mostly based on the morphological variation of the tergal process and, in a few cases, on the genitalia (Azevedo 2000, 2001; Alencar and Azevedo 2006, 2008; Redighieri and Azevedo 2006; Colombo and Azevedo 2016; Colombo et al. 2018). Most of the morphological approaches to *Dissomphalus* species diagnosis use genitalia structure (Mugrabi and Azevedo 2013, 2016; Brito and Azevedo 2017) but a few species are delimited by characters other than genitalia. One of these cases involves two species of the *ulceratus* species-group, *D. concavatus* Azevedo, 1999 and *D. rectilineus* Azevedo, 1999, which are separated by the shape of the hypopygium, but with indistinguishable genitalia (Azevedo 1999; Redighieri and Azevedo 2006). Redighieri and Azevedo (2006) had mentioned that some specimens of *D. rectilineus* resembled *D. concavatus*, with the posterior margin of the hypopygium slightly incurved, the paramere with the dorsal margin well developed, and the apex with four small, rounded teeth. Nevertheless, little was done to solve the ambiguity of identification of these specimens. The authors stated the need for additional work in this group to better understand its diversity. The use of integrative analyses, including DNA sequencing of the mitochondrial gene cytochrome oxidase I (COI) has been useful to identify cryptic species in Hymenoptera (Griffiths et al. 2001; Hebert et al. 2003; Haine et al. 2006; Smith et al. 2009; Santos et al. 2011; Veijalainen et al. 2011; Gebiola et al. 2012; Williams et al. 2012). Thus, we used COI sequences and hypopygium structure to evaluate the species limits of *D. rectilineus* and *D. concavatus* from the Atlantic rainforest, and to understand if those variations hold any utility for identification of these species in the group.

## Material and methods

**Figure 1.**
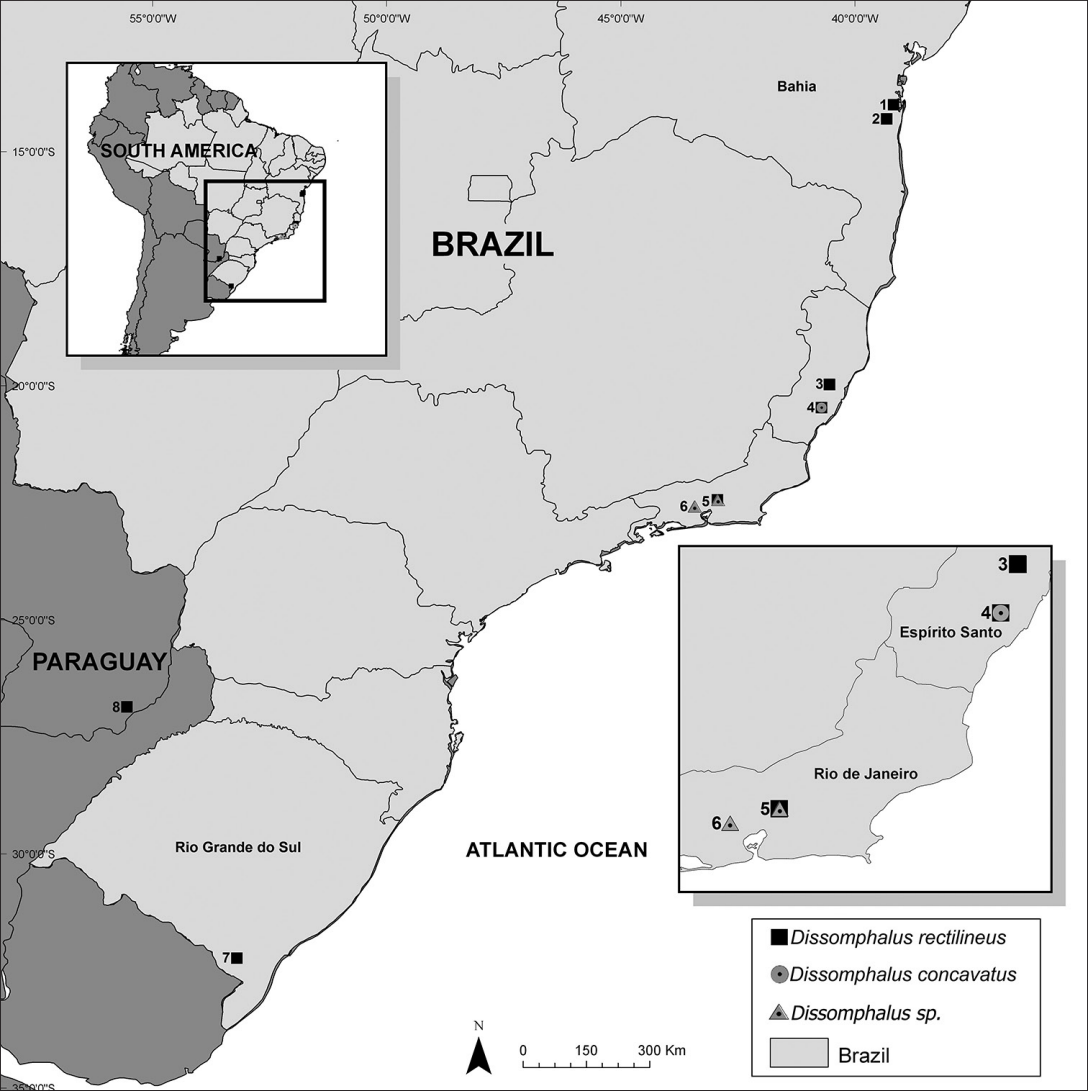
Locations of the samples in Brazil and Paraguay (see Appendix I for geographic coordinates).

From a set of 156 individuals identified by the genitalia and hypopygium shape as *D. rectilineus* and *D. concavatus*, we successfully obtained sequences of 29 individuals (25 *D. rectilineus* and four *D. concavatus*) from six localities in Brazil and one in Paraguay (Fig. 1). These specimens are deposited in the Entomological Collection of Universidade Federal do Espírito Santo (UFES), Vitória, Brazil and sequences are deposited in GenBank at www.ncbi.nlm.nih.gov (see Appendix I for details). As an outgroup we used sequences of three species of *Dissomphalus* from Thailand which were available in our lab from previous studies: *D. thaianus* Terayama, 2001 (T1097); *D. wusheanus* Terayama, 2001 (T2810); and *D. chiangmaiensis* Terayama, 2001 (T3104).

We removed the hypopygium from the genitalia and took at least 20 photographs of each at 9.2 × magnification with the in EntoVision system (GTVision), then compiled the images in the Helicon Focus v5.3.7. The map was generated in the program QGIS 2.18.18.

We obtained genomic DNA from the metasoma or genitalia using the DNA MACHEREY-NAGEL NucleoSpin Tissue kit following the maker’s protocol, with a final suspension volume of 40 µl. Since mini-barcodes are short informative regions of COI that are useful for degraded DNA samples, as obtained from pinned-specimens from museums (Hajibabaei and McKenna 2012; Leray et al. 2013; Tucker et al. 2015; Tucker and Sharkey 2016), we amplified the 340 bp of the final portion of the mitochondrial gene cytochrome oxidase I (mini-COI) by PCR using the primers HCO 2198 designed by Folmer et al. (1994) and AP-L-2176F designed by Pedersen (1996). For the PCR reactions we used a ABI Veriti Thermal Cycler in mixtures containing 3 µl of genomic DNA, 2.5 µl of 10× Taq buffer, 5 mM dNTP mix, 75 mM MgCl_2_, 2.5 mM of each primer, 1U Platinum Taq DNA Polymerase (Invitrogen, Inc.), and distilled water to bring the final volume of 25 µl. The PCR profile consisted of initial denaturation at 94 °C for 5 min, followed by 40 cycles of denaturation at 94 °C for 45 secs, annealing at 47 °C for 45 secs, extension at 72 °C for 45 secs, and a final extension at 72 °C for 5 min. In order to improve the success of PCR amplifications, a second PCR reaction was performed using 0.5 µl of the first PCR product as template, reducing to 30 cycles of thermal cycling. We detected the success of the amplifications on the 2% TBE agarose gel. The PCR products were purified using an ExoSAP-IT kit (USB Corporation) and the DNA was sequenced with an ABI3700 Genetic Analyser (Applied Biosystems, Foster City, CA) using the BigDye Terminator protocol (Applied Biosystems) at the Núcleo de Genética Aplicada à Conservação da Biodiversidade (NGACB) in UFES. Sequences were checked for the correct loci amplification and taxonomy using the Basic Local Alignment Tool (BLAST; Altschul et al. 1990) from GenBank database. Sequences were aligned using MEGA v5.05 (Tamura et al. 2011) using the ClustalWv2.0 algorithm (Larkin et al. 2007). The number of haplotypes (nH), the number of polymorphic sites (s), haplotype diversity (HD) and the nucleotide diversity (π) were estimated using the program DnaSP v5.0 (Librado and Rozas 2009). The genetic divergences between and within groups/clades were calculated using the Tamura and Nei model, with gamma correction and tested with 10,000 permutations using MEGA v5.0 (Tamura et al. 2011). The phylogenetic analyses were inferred using Bayesian Inference (BI) and Maximum-Likelihood (ML). The evolutionary model was determined by JModeltest v0.1 (Posada 2008). BI trees were generated using the software MrBAYES 3.1.2 (Ronquist and Huelsenbeck 2003). The Markov chain was conducted with three million generations with burn-in of 25%, sampling every hundred generations using the evolutionary model proposed by JModeltest. Statistical branch support was obtained through posterior probabilities (PP), and the reliability of the clades was accepted according to the proposal by Hillis and Bull (1993), as following: strong (> 0.95) and moderate (0.85–0.95). The ML analyzes were generated by the PHYML 3.0 platform (Guindon et al. 2010), with 100,000 generations and the evolutionary model best suited calculated by JModeltest. The best evolutionary model for the data matrix using the Akaike correction was GTR + G (Gamma = 0.4510; frequencies of nucleotide bases A = 0.3694, C = 0.1092, G = 0.1485 and T = 0.3728). For the BIC criterion, the best evolutionary model was HKY + G, (transcription rate / transversion = 1.0044, the parameter gamma = 0.3890 and the frequency of nucleotide bases were A = 0.4088, C = 0.0706, G = 0.1273 and T = 0.3933). Branch support was obtained through bootstrapping (BT), and the reliability of the Rambclades was accepted following Hillis and Bull (1993) as follows: strong (> 70%) and moderate (50–70%). The phylogenetic trees were edited with FIGTREE v1.4.2 (http://tree.bio.ed.ac.uk/software/figtree/).

## Results

**Figure 2.**
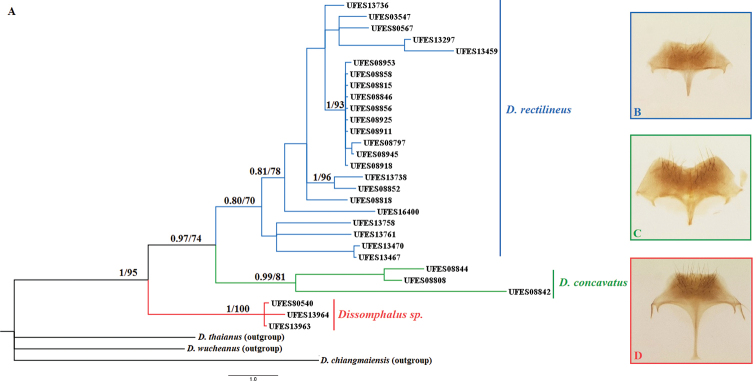
**A** bayesian consensus tree generated from the 304-bp COI from 29 representatives of the species complex. Posterior probabilities (PP) and bootstrap (BT) indicated above branches. The species *D. thaianus*, *D. wusheanus* and *D. chiangmaiensis* were used as outgroups to root the tree **B–D** hypopygium magnified 9.2×, corresponding to each clade.

The 29 successfully amplified COI sequences with 304 bp (18.6%) generated 16 haplotypes that were locality-specific (Appendix I). The ML and BI phylogenetic trees showed a similar topology (Fig. 2A) recovering three well-supported clades (BT = 70–100, PP = 0.8–1). The within-clade genetic distances varied from 1.3% for clade III, 7.7% for clade I and 13.4% for clade II, while the between-clade divergences varied from 19 to 20%. We calculated the genetic divergence of 28% between the ingroup vs. the outgroup. Twenty-three out of 29 sequences recovered clade I, with specimens from Bahia (BA) to Rio Grande do Sul (RS) in Brazil and one location in Paraguay, showing 16 haplotypes. Three sequences of specimens from Nova Iguaçu (RJ) recovered clade III with two haplotypes; while three sequences of specimens from Alfredo Chaves (ES) recovered clade II, each one with a distinct haplotype (Fig. 2, Appendix I).

We detected three distinct hypopygium shapes, each of them associated with a clade (Fig. 2B–D). The hypopygium associated with clade I showed a straight posterior margin, and a narrower base of the stalk, which is similar to that described by Azevedo (1999) for *D. rectilineus*. The hypopygium associated with clade II is much more robust and angular than the others, with the posterior margin clearly incurved, which is associated with *D. concavatus*. The hypopygium associated with clade III was more elongated than the other two, and the trapezoidal plate has the stalks extremely long with the end enlarged, distinct from any other hypopygium described in this group.

## Discussion

Our phylogenetic analysis showed that specimens originally identified as *D. rectilineus* were arranged over two well-supported monophyletic groups (clades I and III) with 19% of divergence. Clade I grouped 23 individuals from Bahia to Rio Grande do Sul in Brazil and in Paraguay, with an intraclade divergence of 7.7%, while clade III grouped three specimens from Espírito Santo with an intraclade divergence of 1.3%. Three specimens originally identified as *D. concavatus* were grouped as a single monophyletic lineage (clade II), which led us to assume that each clade corresponds to a well-supported species, with genetic distance of 19–20%, consistent with species-level divergence. The genetic divergence values of COI herein recovered for these closely related species were surprisingly high compared to the values mentioned by Hebert et al. (2003), but similar to those found in other parasitoid wasp studies, that registered variation from 4.4% to 26% (Haine et al. 2006; Moe and Weiblen 2010; Sun et al. 2011; Veijalainen et al. 2011; Vink et al. 2012).

Distinguishing between *D. rectilineus* and *D. concavatus* is difficult due to their sympatric distribution and shared morphological characters which overlap in several important structures. *Dissomphalus concavatus* was described with the posterior margin of the hypopygium incurved and the base of the stalk widened, whereas *D. rectilineus* has a posterior margin straight and a narrower base of the stalk (Azevedo 1999). The *ulceratus* species-group was diagnosed by having the basal portion of the dorsal margin of the paramere well developed and is currently composed of four species in the central-eastern region of South America, showing some sympatry in Brazil: *D. concavatus* in Espírito Santo, São Paulo and Paraná states and Distrito Federal; *D. rectilineus* in Espírito Santo, Rio de Janeiro, São Paulo and Paraná states and *D. dentiformis* Azevedo, 1999 in Distrito Federal; and *D. ulceratus* Evans, 1969 was recorded in Tucuman, Argentina. Nevertheless, even though we identified the specimens herein as *D. rectilineus* and *D. concavatus*, our analysis indicated three well-defined, distinct monophyletic groups, with high divergence, each of them with unique and exclusive polymorphisms.

Thus, we are inclined to assume that clade III representatives were consistent with a new species of the *ulceratus* species-group in Espírito Santo, with the hypopygium characteristics distinct from any other recognized forms of hypopygium described for *Dissomphalus*, mainly defined by having the posterior margin straight, and stalk with the base very wide and long. Associated with high genetic divergence, there is enough evidence from morphological and genetic analyses to support this clade as a distinct species within the analyzed specimens. This species belongs to the *ulceratus* species-group because of the morphology of the tergal process (see Azevedo 1999), but its genitalia and the general body morphology is different from all species of this group; it will be formally described in a future taxonomic revision.

The hypopygium helps to evert the genitalia through inserted muscles from the tip of the stalk to the tip side of the median stalk (Schulmeister 2001). The bristles on the hypopygium seem to have a sensorial function, used to determine the position of the genitalia (Austin and Dowton 2000).

The hypopygium structure allowed the distinction of specimens as either *D. rectilineus* or *D. concavatus* (Azevedo 1999), but Redighieri and Azevedo (2006) mentioned the posterior margin of hypopygium was slightly incurved for some specimens of *D. rectilineus* from Espírito Santo, which coincides with the structure of the hypopygium described for clade III. In other studies, the *Dissomphalus* species (including new descriptions) were defined by the posterior margin of the plate but a few were associated with the length of the median stalk, the base width of the median stalk and the degree of concavity (Azevedo 1999; Azevedo 2003; Redighieri and Azevedo 2006).

In this study, three individuals from Alfredo Chaves (ES) were not previously identified by hypopygium (UFES 08856, UFES 08925 and UFES 08852) but due to the robustness of the clades, we were able to classify them undoubtedly as *D. rectilineus*, showing the importance of integrative analyses for specimens’ identification in the group. The implementation of DNA barcoding is helping to solve taxonomic problems and showing that species diversity is still underestimated (Griffiths et al. 2001; Hebert et al. 2003; Haine et al. 2006; Smith et al. 2009; Santos et al. 2011; Veijalainen et al. 2011; Carolan et al. 2012; Gebiola et al. 2012; Williams et al. 2012; present study). Due to the sharp decline in global biodiversity, methods of analysis and species identification that show, more reliably, the richness of biodiversity are of great importance. The integration of molecular and morphological methods can help to accelerate the biodiversity inventory and facilitate the identification of cryptic species, and considering museum animals, the use of mini-barcodes is a very important tool because it allows for the extraction of valuable information using degraded DNA.

## Conclusions

Based on genetic divergence data, we concluded that *Dissomphalus concavatus* and *D. rectilineus* are distinct species, and that the delimitation based on hypopygium morphology proposed by Azevedo (1999) is cladistically supported, so that the hypopygium can be used as a diagnostic character when applicable. We also found data showing high genetic divergence (around 19–20%) indicating species-level divergence in the group, whereas 1.3–13.4% was registered as within species-level divergence. Such a finding stimulates future investigations in the group because its biodiversity appears to be underestimated. Additionally, our results support the occurrence of an unnamed species for the *ulceratus* species-group, distinct from the four described species.

## Appendix I

**Table:**

Species	Voucher Collection Number	Original ID	Hypopygium shape (Fig. 1)	Municipality	State/Country	Number in Fig. 1	Geographic coordinate	COI seq. Length	COI Haplotype	Genbank Number
*D. rectilineus*	UFES 13736	*D. rectilineus*	B	Santa Teresa	Espirito Santo / Brazil	3	19°58.62'S, 40°32.17'W	304	1	MT602017
	UFES 13738	*D. rectilineus*	B	Santa Teresa	Espirito Santo / Brazil	3	19°58.62'S, 40°32.17'W	304	2	MT602018
	UFES 13761	*D. rectilineus*	B	Santa Teresa	Espirito Santo / Brazil	3	19°58.62'S, 40°32.17'W	304	15	MT602037
	UFES 13758	*D. rectilineus*	B	Santa Teresa	Espirito Santo / Brazil	3	19°58.62'S, 40°32.17'W	304	14	MT602036
	UFES 08953	*D. rectilineus*	B	Alfredo Chaves	Espirito Santo / Brazil	4	20°27.88'S, 40°42.58'W	304	5	MT602021
	UFES 08858	*D. rectilineus*	B	Alfredo Chaves	Espirito Santo / Brazil	4	20°27.88'S, 40°42.58'W	304	6	MT602022
	UFES 08815	*D. rectilineus*	B	Alfredo Chaves	Espirito Santo / Brazil	4	20°27.88'S, 40°42.58'W	304	6	MT602023
	UFES 08856	*D. rectilineus**	-	Alfredo Chaves	Espirito Santo / Brazil	4	20°27.88'S, 40°42.58'W	304	6	MT602026
	UFES 08925	*D. rectilineus**	-	Alfredo Chaves	Espirito Santo / Brazil	4	20°27.88'S, 40°42.58'W	304	6	MT602027
	UFES 08852	*D. rectilineus**	-	Alfredo Chaves	Espirito Santo / Brazil	4	20°27.88'S, 40°42.58'W	304	7	MT602024
	UFES 08846	*D. rectilineus*	B	Alfredo Chaves	Espirito Santo / Brazil	4	20°27.88'S, 40°42.58'W	304	6	MT602025
	UFES 08797	*D. rectilineus*	B	Alfredo Chaves	Espirito Santo / Brazil	4	20°27.88'S, 40°42.58'W	304	9	MT602030
	UFES 08818	*D. rectilineus*	B	Alfredo Chaves	Espirito Santo / Brazil	4	20°27.88'S, 40°42.58'W	304	8	MT602028
	UFES 08911	*D. rectilineus*	B	Alfredo Chaves	Espirito Santo / Brazil	4	20°27.88'S, 40°42.58'W	304	6	MT602029
	UFES 08918	*D. rectilineus*	B	Alfredo Chaves	Espirito Santo / Brazil	4	20°27.88'S, 40°42.58'W	304	6	MT602031
	UFES 08945	*D. rectilineus*	B	Alfredo Chaves	Espirito Santo / Brazil	4	20°27.88'S, 40°42.58'W	304	10	MT602032
	UFES 16400	*D. rectilineus*	B	Arroio Grande	Rio Grande do Sul / Brazil	7	32°13.37'S, 53°11.95'W	304	11	MT602033
	UFES 13459	*D. rectilineus*	B	Ubaitiba	Bahia / Brazil	2	14°18'S, 39°19'W	304	13	MT602035
	UFES 13297	*D. rectilineus*	B	Camamu	Bahia / Brazil	1	14°00'S, 39°10'W	304	12	MT602034
	UFES 13467	*D. rectilineus*	B	Ubaitiba	Bahia / Brazil	2	14°18'S, 39°19'W	304	17	MT602039
	UFES 13470	*D. rectilineus*	B	Ubaitiba	Bahia / Brazil	2	14°18'S, 39°19'W	304	16	MT602038
	IBES 03547	*D. rectilineus*	B	–	Paraguay	8	26°51.6'S, 55°32.7'W	304	3	MT602019
	UFES 80567	*D. rectilineus*	B	Rio de Janeiro	Rio de Janeiro / Brazil	5	22°26'S, 42°56'W	304	4	MT602020
*D. concavatus*	UFES 08808	*D. concavatus*	C	Alfredo Chaves	Espirito Santo / Brazil	4	20°27.88'S, 40°42.58'W	304	20	MT602042
	UFES 08842	*D. concavatus*	C	Alfredo Chaves	Espirito Santo / Brazil	4	20°27.88'S, 40°42.58'W	304	19	MT602041
	UFES 08844	*D. concavatus*	C	Alfredo Chaves	Espirito Santo / Brazil	4	20°27.88'S, 40°42.58'W	304	18	MT602040
*Dissomphalus* sp	UFES 80540	*D. rectilineus*	D	Rio de Janeiro	Rio de Janeiro / Brazil	5	22°26'S, 42°56'W	304	21	MT602043
	UFES 13964	*D. rectilineus*	D	Nova Iguaçu	Rio de Janeiro / Brazil	6	22°34.62'S, 43°26.08'W	304	22	MT602044
	UFES 13963	*D. rectilineus*	D	Nova Iguaçu	Rio de Janeiro / Brazil	6	22°34.62'S, 43°26.08'W	304	23	MT602045

* Specimens without hypopigium, species defined based on molecular data identification.
